# Antimycotic Ciclopirox Olamine in the Diabetic Environment Promotes Angiogenesis and Enhances Wound Healing

**DOI:** 10.1371/journal.pone.0027844

**Published:** 2011-11-18

**Authors:** Sae Hee Ko, Allison Nauta, Shane D. Morrison, Hongyan Zhou, Andrew Zimmermann, Geoffrey C. Gurtner, Sheng Ding, Michael T. Longaker

**Affiliations:** 1 Hagey Laboratory for Regenerative Medicine, Division of Plastic and Reconstructive Surgery, Department of Surgery, Stanford University School of Medicine, Stanford, California, United States of America; 2 Department of Surgery, University of Pittsburgh School of Medicine, Pittsburgh, Pennsylvania, United States of America; 3 Department of Surgery, Georgetown University School of Medicine, Washington, D.C., United States of America; 4 Stanford University School of Medicine, Stanford, California, United States of America; 5 Gladstone Institute of Cardiovascular Disease, University of California San Francisco Mission Bay Campus, San Francisco, California, United States of America; 6 Stanford University, Stanford, California, United States of America; 7 Institute of Stem Cell Biology and Regenerative Medicine, Stanford University School of Medicine, Stanford, California, United States of America; Medical College of Georgia, United States of America

## Abstract

Diabetic wounds remain a major medical challenge with often disappointing outcomes despite the best available care. An impaired response to tissue hypoxia and insufficient angiogenesis are major factors responsible for poor healing in diabetic wounds. Here we show that the antimycotic drug ciclopirox olamine (CPX) can induce therapeutic angiogenesis in diabetic wounds. Treatment with CPX *in vitro* led to upregulation of multiple angiogenic genes and increased availability of HIF-1α. Using an excisional wound splinting model in diabetic mice, we showed that serial topical treatment with CPX enhanced wound healing compared to vehicle control treatment, with significantly accelerated wound closure, increased angiogenesis, and increased dermal cellularity. These findings offer a promising new topical pharmacologic therapy for the treatment of diabetic wounds.

## Introduction

Diabetic wounds are an area of increasing concern with the epidemic proportions of diabetes incidence. In 2003, the number of diabetic patients worldwide was already estimated at 197 million, and this number is projected to increase to 366 million by 2030 due to increased longevity [1]. Over 23 million people, or 7.8% of the US population, suffer from diabetes, and up to 25% of all diabetics are estimated to develop a diabetic foot ulcer [2]. In 2004, approximately 71,000 nontraumatic lower-limb amputations were performed in diabetic patients[3]. The amputation of a limb lends significant impact not only on patient's quality of life but also on patient mortality, with 5-year survival rate after a lower-limb amputation of only 50% [2]. Nonhealing lower extremity diabetic ulcers account for approximately 25–50% of all hospital admissions in the diabetic population and are responsible for the majority of resultant amputations [4].

A critical stimulus for normal wound healing is relative hypoxia, and an impaired response to hypoxia could contribute to impaired wound healing in diabetes [5]. Hypoxia-inducible factor (HIF)-1, which is a heterodimeric transcription factor complex consisting of hypoxia-stabilized α-subunit (HIF-1α) and a constitutively expressed β-subunit (HIF-1β), functions as a central regulator of oxygen homeostasis. Under hypoxic conditions, stabilized HIF-1α translocates to the nucleus, where it dimerizes with HIF-1β and binds to a hypoxia response element (HRE) present on multiple genes that are essential for cell survival during hypoxia [6]. HIF-1α is critical for expression of multiple angiogenic growth factors, cell motility, and recruitment of endothelial progenitor cells [7]–[9].

Prolyl hydroxylase domain (PHD)-2 is the key oxygen-dependent negative regulator of the protein stability of HIF-1α [10]. Proline hydroxylation serves as a critical step in determining the biological half-life of HIF-1α, and transient silencing PHD-2 results in normoxic stabilization of HIF-1α [11]. In addition, recent evidence indicates that PHD-2 has an additional key physiological role regulating angiogenesis through a HIF-independent mechanism [10]. Thus, PHD-2 is a promising target for inducing therapeutic angiogenesis for the treatment of chronic diabetic wounds.

While recombinant angiogenic growth factors and gene therapy have been proposed as viable treatment modalities for therapeutic angiogenesis, these modalities are hindered by many barriers to mainstream medicine including safety concerns and high cost. A pharmaceutical approach may be the most practical and beneficial approach in the present considering its safety, cost, and ease of application. The antimycotic drug ciclopirox olamine (CPX) is a bidentate iron chelator capable of inhibiting PHD-2 and activating HIF-1α [12]. In this article, we evaluated whether the antimycotic ciclopirox olamine (CPX) can be used as a topical treatment for chronic diabetic wounds using a diabetic mouse wound model. Our novel findings using CPX, an already Federal Drug Administration (FDA) approved drug with a safe side effect profile, offer a promising direction for the pharmacologic treatment of diabetic wounds.

## Results

### CPX is a potent inducer of HIF-1a stabilization and VEGF expression

**Figure 1:**
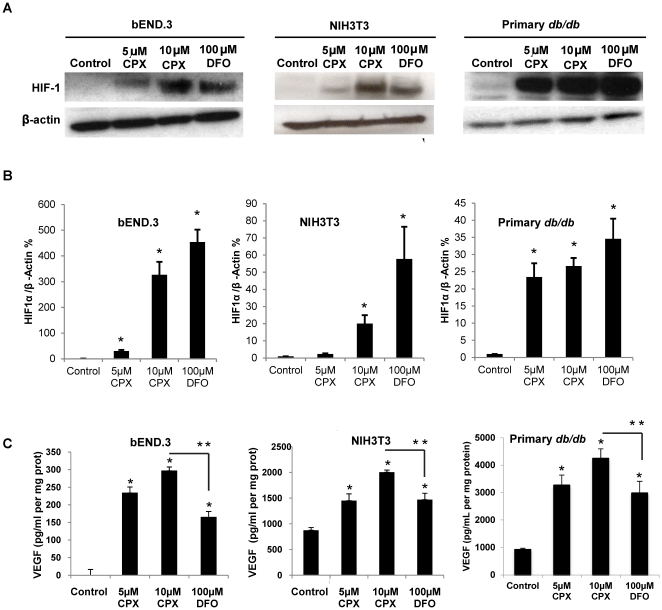
Effect of CPX treatment *in vitro*. (**A**) Treatment with 5–10 µM CPX for 24 hours on bEND.3 endothelial cell line, NIH3T3 cell line, and primary diabetic fibroblasts stimulated HIF-1α activation in a dose-dependent manner (**B**) Densiometry data showing pixel density of Western blot data calculated using ImageJ software. Pixel density is expressed as a ratio to β-actin and normalized to untreated cells. Astericks indicate statistically significant increase in protein concentration relative to untreated controls. (*p<0.05) (**C**) Conditioned media of cells treated with 5–10 µM CPX for 24 hours led to a significant increase in VEGF expression as detected by ELISA in bEND.3, NIH3T3, and diabetic primary fibroblast cells. (*p<0.05).

The effects of CPX on HIF-1α induction in mouse endothelial cell line bEND.3 and in mouse fibroblast cell line NIH3T3 were compared with deferoxamine (DFO), an iron-chelator well known for HIF-1-inducing capabilities. The two cell lines were chosen to simulate the types of cells (fibroblasts and endothelials cells) that are likely to be affected by the pharmacologic treatment with CPX in a cutaneous wound. After 24 hours of exposure to CPX, there was a robust HIF-1α stabilization in both cell types in a dose-dependent manner as detected by Western Blot (**Figure 1A**). CPX was more potent than DFO in inducing HIF-1α, with 10 µM CPX inducing comparable HIF-1α stabilization response compared to 10-fold higher 100 µM DFO. Primary diabetic fibroblasts responded similarly with marked induction of HIF-1α after 24 hours of exposure to CPX (**Figure 1A, B**). We next investigated whether CPX also induces the endogenous HIF-1 target gene VEGF, which is an essential mediator of angiogenesis. We confirmed that treatment with the same range of CPX concentrations that increased HIF-1α led to a significant accumulation of VEGF in the supernatant of both types of treated cell line cultures and primary diabetic cell culture compared to the untreated controls in a dose-dependent manner as measured by ELISA (**Figure 1C**). CPX was also more potent than DFO in inducing VEGF protein production. (**Figure 1C**).

### CPX induces multiple angiogenic genes

**Figure 2:**
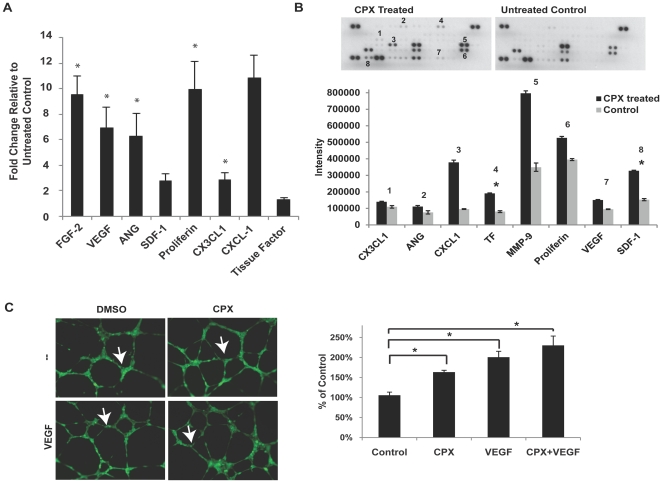
Downstream effects of CPX treatment *in vitro*. (**A**) QRT-PCR analysis on NIH3T3 cells treated with 10 µM CPX for 24 hours versus untreated control. There were 3 to10-fold increases in the mRNA expression of multiple angiogenesis-related genes relative to that of untreated control cells. Asterisks indicate *p<0.05. (**B**) Angiogenesis antibody array using condition media from NIH3T3 cells treated with 10 µM CPX for 24 hours versus condition media from untreated control cells. Membrane images of antibody array treated with condition media of treated and control cells (top). Specific proteins captured on the array are labeled 1 to 8. Semi-quantitative profiles of proteins 1 to 8 on the angiogenesis antibody array using reverse image scanning densitometry (bottom). There was a significant increase in the expression of multiple angiogenic proteins by NIH3T3 cells treated with CPX compared to untreated control cells. Asterisks indicate *p<0.05. (**C**) The ability of CPX (10 µM) in inducing *in vitro* tubule formation with or without VEGF (10 ng/ml) treatment. Representative cell images showing field of view as indicated (left). The angiogenic effect was quantified by counting the number of branch points per field of view (right). Endothelial cells treated with CPX (10 µM) increased the number of branch point cells with or without concomitant VEGF (10 ng/ml) treatment compared to those cells treated with DMSO control (40X). White arrows indicate examples of branch points (*p<0.05).

Given our observation of increased HIF-1α and VEGF with CPX treatment, we further explored the potential of CPX as a therapeutic agent for angiogenesis by evaluating the expression levels of other common angiogenesis-related genes. We next chose to determine the effect of treatment with 10 µM CPX for 24 hours in NIH3T3 cells in comparison to untreated cells on the multiple other angiogenesis-related genes' mRNA levels using quantitative, real-time-polymerase chain reaction (QRT-PCR). This experiment established that there was significant upregulation of multiple other angiogenic genes (**Figure 2A**). These included endogenous genes under direct regulation by HIF-1α such as fibroblast growth factor (FGF)-2 and VEGF. In addition, there was also 3 to 10-fold increase in the expression of multiple angiogenic cytokines that are not known to be under direct regulation HIF-1α including angiogenin (ANG), CX3CL1, and proliferin. Elevated angiogenic gene expression was confirmed at the protein level using the angiogenesis antibody array, where multiple common angiogenesis-related proteins were over-expressed in the supernatant of the treated cells compared to that of untreated control cells (**Figure 2B**). Some differences were noted between the levels of mRNA expression and protein expression for genes, which may be attributed to timing differences between gene and protein expression and possible post-transcriptional mechanisms affecting mRNA stability and protein degradation. Nonetheless, there was a clear trend toward elevated angiogenic gene expression in cells treated with CPX at both mRNA and protein levels.

### CPX induces angiogenesis in vitro

Given that CPX treatment strongly induces multiple angiogenic genes, we next examined the effect of CPX on the vasculature using a short-term *in vitro* assay using human umbilical vein endothelial cells (HUVEC cells). This assay evaluates the propensity of primary endothelial cells to sprout and form three-dimensional structures resembling capillaries in response to appropriate cues. To achieve a statistically significant morphometric analysis of capillary density, blood vessel branching points were counted in nine high power fields. Compared to treatment with dimethyl sulfoxide (DMSO), treatment with 10 µM CPX significantly increased the number of branch points per field of view, indicating elevated angiogenesis. When 10 ng/ml VEGF was co-administered with CPX, there was slightly increased tubule formation by cells compared to cells treated with VEGF alone (**Figure 2C**).

### Topical CPX application Enhances Wound Healing and Angiogenesis in Diabetic Mice

Because CPX strongly induces angiogenic genes and endothelial tubule formation *in vitro*, we next evaluated the potential therapeutic efficacy of CPX *in vivo* using a humanized wound healing model in leptin deficient type II diabetic *db/db* mice. By stenting the wounds with silicone rings, mouse wounds heal primarily by granulation tissue deposition similar to humans, rather than by contraction which occurs in conventional mouse wound models [13].

**Figure 3:**
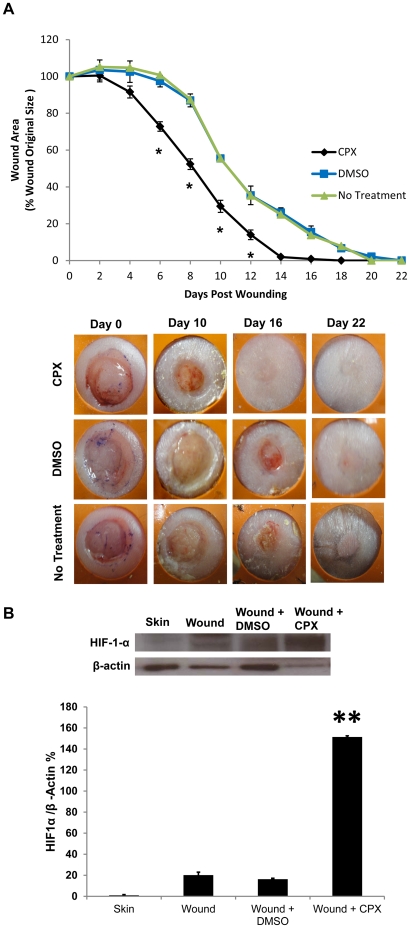
Topical CPX enhanced wound healing in diabetic mice. Full-thickness skin wounds on diabetic mice were treated topically with 50 mM CPX, DMSO as vehicle control, and no treatment every other day until wound closure. (**A**) Wound closure rate over time. There was a significant acceleration of wound healing in the CPX treatment group (n = 10; *p<0.05) (top). Representative photographs of wounds treated with DMSO vehicle control, no treatment, or 50 mM CPX (bottom). Treatment with CPX healed wounds significantly faster with increased vascularity (grossly represented in red color) in the wound bed compared to vehicle control. (**B**) Western blot showing statistically significant HIF-1α expression in tissue lysates harvested from wounds treated with CPX compared to those harvested from unwounded untreated skin, untreated wounded skin, and vehicle-treated wounded skin. (*p<0.01)

**Figure 4:**
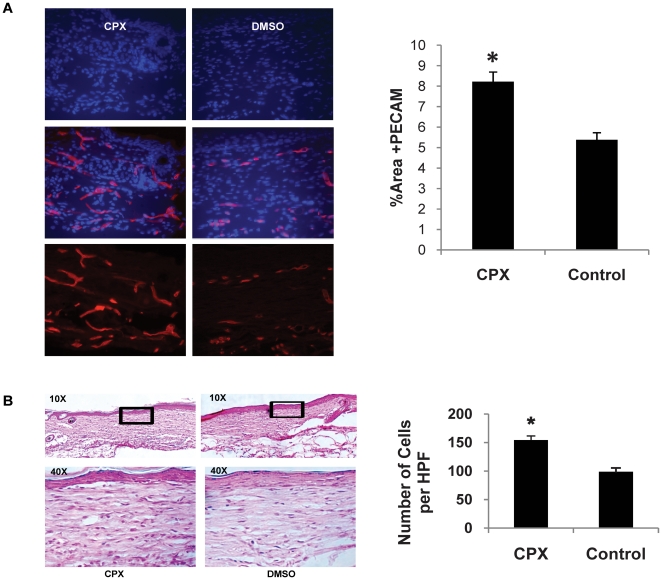
Histological analysis of wounded skin. (**A**) Effects of CPX on wound vascularity. Wound sections were stained with an anti-CD31 antibody and detected with Fluor 594 (red). Software-assisted quantification of vessel density in the entire area of residual wounds at day 22, as percent fluorescence. Wounds from CPX-treated mice had significantly higher vessel density compared with that from DMSO-treated mice (*p<0.05). (**B**) Hematoxylin and Eosin stains of wound tissue after full closure at 22 days post-wounding (left). CPX-treated wounds have higher dermal cellularity in the wound bed compared to DMSO-treated control wounds. The black box indicates the area of healed wound depicted under high power view. Quantification of cellularity in the wound by counting total cell number per high-powered field views (10×40) (right).

Serial topical application every other day with 50 mM CPX on the wounds of diabetic mice significantly enhanced the wound healing response compared to the DMSO treated wounds. Fifty mM dosing was chosen to approximate the 1–8% CPX concentration currently available as dermal cream or nail lacquer preparations for clinical usage. Topical treatment with CPX accelerated wound closure compared to controls, with complete closure occurring by day 14 post-wounding in CPX-treated mice versus day 22 in DMSO vehicle-treated mice (*p<0.05) (**Figure 3A**). No significant systemic side effects such as weight loss or local side effects such as increased edema or allergic reaction were noted in CPX-treated mice. Interestingly, CPX-treated wound beds appeared strikingly more red at day 10 post-wounding compared to DMSO-treated control wounds due to the increased angiogenesis in the CPX-treated wound beds, and this was associated with an early re-epithelialization of the CPX-treated wounds compared to control wounds (**Figure 3A**). The gross observation of increased wound vascular density by CPX was confirmed by histology, with evidence of enhanced angiogenesis with a statistically significant 53% increase in the number of blood vessels as measured by the number of CD31-positive cells in the wound bed of CPX-treated animals relative to the number of blood vessels in the wound bed of control animals (*p<0.05). (**Figure 4A**).

HIF-1α protein concentration was increased in skin tissue lysates of wounds treated with CPX through postoperative day 4. Western blot for HIF-1α demonstrate a significant increase in protein concentration in wounds treated with CPX versus unwounded untreated skin, wounded untreated skin, and vehicle treated wounded skin (*p<0.01). (**Figure 3B**).

Histological analysis showed increased capillary density in wounded skin treated with CPX. Capillary density was quantified by software-assisted analysis of pixel number (Adobe Photoshop software) per section of the wound bed after immunofluorescence staining for endothelial cells with an anti-CD31 antibody. (**Figure 4A**) Therapeutic angiogenesis induced by CPX in the diabetic wounds resulted in a robust increase in the dermal cellularity of healed wounds compared to DMSO-treated control wounds. (**Figure 4B**).

## Discussion

Diabetic wounds remain a major challenge in current clinical practice with often disappointing outcomes despite best available care. Only two-thirds eventually heal, and up to 28% may result in some form of amputation [14]–[16]. Insufficient angiogenesis has been implicated in the abnormal wound healing that leads to diabetic skin ulcers [17]. In this report, we show that the antimycotic drug CPX is a potent inducer of therapeutic angiogenesis via HIF-1α stabilization. Using a humanized diabetic wound model, we demonstrate that serial topical application of CPX caused significant acceleration of wound healing by inducing improved angiogenic response.

Currently, there is little to no evidence to support the use of topically applied products in the treatment of diabetic foot ulcers in routine clinical practice [18]. Despite the relatively large number of studies of growth factors, recombinant platelet derived growth factor BB (PDGF-BB) is the only agent approved by FDA for clinical use in diabetic ulcers, and outcomes from the use of this agent have been disappointing [19]. Because pathogenesis of diabetic ulcers is complex, application of a single growth factor such as PDGF is likely an inadequate approach to accelerating the repair of chronic diabetic wounds. Many growth factors essential for wound healing, including platelet derived growth factor (PDGF), fibroblast derived growth factor 2 (FGF-2), vascular endothelial growth factor (VEGF), and stromal cell derived factor-1 alpha (SDF-1α), are reduced in diabetic wounds [20]–[23]. Diminished production of HIF-1α and its target genes such as VEGF are thought to contribute to insufficient angiogenesis that results in impaired tissue repair in diabetes [21], [23], [24]. Therefore, upregulation of the upstream transcription factor HIF-1α is potentially a more effective therapeutic strategy compared to single growth factor application. Furthermore, considering the safety, costs, and ease of application, a pharmaceutical approach to increasing HIF-1α would be the most practical and beneficial solution.

In this study, a leptin deficient type II diabetic mouse model was used to demonstrate positive effects of CPX on angiogenesis and wound healing. Although some recent literature suggests that a polygenic model of diabetes may better approximate the type II diabetic human phenotype [25], the literature supporting the use of a single model is limited, and all small animal models of diabetes have limitations. The *db/db* leptin deficient mouse model was chosen for these experiments due to its preeminence in the literature as a well-established, reproducible model of diabetic wound healing.[26]

CPX has previously been shown in the literature to induce angiogenesis through the stabilization of HIF-1α and resultant VEGF induction, both *in vitro* and *in vivo*.[12] Using a diabetic excisional wound healing model, we confirmed these findings and showed that CPX is capable of stabilizing HIF-1α and activating many endogenous target genes important for wound healing such as VEGF, FGF-2, and SDF-1. CPX was first developed to treat fungal skin infections and vaginal candidiasis and currently has well established efficacy for these indications [27]. Although its exact mechanism of action is unknown, the high affinity of CPX for trivalent metal cations, resulting in inhibition of the metal-dependent enzymes that are responsible for the degradation of peroxides within the fungal cell, is thought to be the major determinant of its antimycotic activity [27]. CPX is also a potent iron chelator, and therefore can also indirectly inhibit PHD-2, the key upstream negative regulatory gene in HIF-1 pathway that requires iron as a cofactor [12], [28]. Prolyl hydroxylase domain-2 (PHD-2) has additional HIF-independent functions in mediating the angiogenic switch via negative regulation of nuclear factor kappa-light-chain-enhancer of activated B cells (NF-κB). [29] We found that treatment with CPX not only led to increased HIF-1α protein availability and its downstream angiogenic genes but also activated many NF-κB-dependent pro-angiogenic factors including ANG, CX3CL1, CXCL1, and tissue factor. Therefore, pharmacologic inhibition of PHD-2 may be a mechanism by which CPX enhances the angiogenic response in the diabetic environment, and this mechanism may be more effective than gene transfer techniques aimed only at HIF-1α gain-of-function.

Although iron chelators have been shown to decrease inflammation in the wound bed and accelerate wound closure, it is also thought that large doses of these compounds can induce cell death. [30] This dose response is important to consider when designing clinical trials for the use of CPX and other iron chelators in wound healing, as high doses may be more appropriate for the treatment of hyper-proliferative states, such as malignancy.

Increased angiogenesis may allow for increased oxygen and nutrient delivery to developing granulation tissue, which can have other positive effects on wound healing, such as increased migration, decreased apoptosis, and increased cell survival. For example, in addition to allowing increased availability of HIF-1α protein and activating other angiogenic factors in the wound bed, CPX was found to increase dermal cellularity. During the proliferative phase of wound healing, macrophages release factors that stimulate granulation tissue formation. The fibrin clot is replaced by highly vascular tissue made up of many different cell types, including—but not limited to—fibroblasts and myofibroblasts. During remodeling, which can last a protracted period, these cells remain active in the wound, as an avascular and acellular scar develops. [31], [32]

Compared to wild type mice, diabetic mice demonstrate impaired healing for size-matched excisional wounds. Hematoxylin and Eosin stains of fully healed wounds demonstrate increased dermal cellularity at day 22 in CPX treated wound beds, indicating an increase in dermal proliferation and remodeling. This finding suggests that at day 22, wounds treated with CPX are further along in the proliferative and remodeling phases of wound healing than untreated or vehicle control treated wounds.

Our finding that serial topical CPX treatment significantly enhances healing of humanized diabetic wounds has significant clinical implications. Linden *et al.* suggested that CPX may be beneficial for skin wound healing based on the observation that CPX induced angiogenesis in the chicken chorioallantoic membrane [12]. However, to the best of our knowledge, no published studies to date have evaluated the efficacy of CPX in the treatment of diabetic wounds. Another iron chelator, DFO, used systemically in the treatment of chronic iron overload, has been shown to activate HIF-1α and enhance diabetic wound healing when it is applied topically to the wound bed [23]. While CPX and DFO share a similar mechanism of action (i.e. iron depletion), CPX may be a more attractive candidate for pharmacologic therapy for chronic diabetic wounds. CPX has already been shown to be an effective treatment agent for onychomycosis in diabetic patients due to its efficacy, excellent safety profile with extremely low systemic absorption, and ease of use, which has resulted in high patient compliance [33]. Diabetic patients are at increased risk for dermatomycoses and onychomycoses, with approximately one-third of diabetic patients affected [34]. Diabetic patients with onychomycosis have a substantially higher risk of complications such as secondary foot infections, gangrene, and foot ulcers compared with diabetic patients without onychomycosis [33]. Thus, having a product that is both fungicidal and safe to use is beneficial to diabetic patients with chronic wounds. In addition, a treatment that requires serial topical application requires that patient give attention to their wounds daily, which is ideal for diabetic patients who should be examining their feet regularly to monitor ulcer developments. Furthermore, because topical CPX treatment is already well-established for the treatment of onychomycosis and dermatomycoses, the potential for CPX to heal chronic diabetic wounds in humans could be evaluated in clinical trials relatively quickly.

In conclusion, serial topical treatment with the antimycotic drug CPX may be regarded as a promising novel therapeutic treatment for diabetic ulcers. Additional human clinical trials will be required to determine the clinical applicability of CPX treatment to improve wound healing in diabetic patients.

## Materials and Methods

### Cell lines and culture

The mouse fibroblast cells NIH3T3 were cultured in Dubecco's Modified Eagle's Medium (DMEM; high glucose) supplemented with 10% bovine calf serum (Gibco) as described in the American Type Culture Collection protocol. The mouse endothelial cells bEND.3 were cultured in DMEM medium (high glucose) supplemented with 10% fetal bovine serum (Gibco) as described in the ATCC protocol. Human umbilical vein endothelial cells were purchased from Lonza (C2517A). Cells were maintained in culture in the supplier's recommended complete medium (Endothelial Cell Growth Medium-2) at 37°C, 5% CO_2_. The maximum passage number used for experiments was six, and cells were pre-cultured in serum free medium for 24 hr before angiogenesis assay. CPX (5–10 µM) and deferoxamine (DFO; 100 µM) were purchased from Sigma and CPX was prepared in dimethyl sulphoxide (DMSO; Sigma) for indicated experiments.

### Isolation of primary diabetic dermal fibroblasts

Dorsal skin from adult diabetic *db/db* mice (BKS.Cg-*Dock7^m^* +/+ *Lepr^db^*/J, stock #000642; Jackson Laboratories) was excised and used for the isolation of fibroblasts as reported previously [35]. Briefly, harvested skin tissues were washed with Betadine (Fisher Scientific) and PBS, minced, then incubated in a collagenase/dispase (Roche) mixture at 37°C with constant shaking for 3 hours to obtain single cell suspensions. The primary diabetic fibroblasts were cultured in DMEM medium supplemented with fetal bovine serum (Gibco). The primary fibroblasts were passaged twice, then treated with CPX, DMSO, or DFO.

### Vascular endothelial growth factor (VEGF) enzyme-linked immunosorbent assay (ELISA)

Quantikine murine VEGF ELISA kits (R&D Systems) were used according to the manufacturer's protocol. Briefly, VEGF standards (1 to 1000 pg/mL) and samples were placed by pipette into wells coated with antibody specific for mouse VEGF. After a wash, an enzyme-linked polyclonal antibody specific for VEGF was added to the wells. After a second wash, a substrate solution was added. The absorbance of standards and samples was measured spectrophotometrically at 450 nm with a wavelength correction set to 570 nm using a microplate reader. VEGF concentrations were calculated (in pg/mL) with the standard curve via four parameter logistic (4-PL) curve-fit model using GraphPad Prism (GraphPad Software, Inc.), and then adjusted for protein concentrations. Total protein concentrations in each sample were determined by the Bradford assay (Bio-Rad).

### Western blotting

NIH3T3, bEND.3, and primary diabetic fibroblasts were exposed to 5–10 µM CPX or 100 µM DFO for 24 hours as specified. Cells were lysed in urea lysis buffer (9 M urea, 75 mM Tris [pH 7.5], 150 mM ß-mercaptoethanol). Cells were sonicated briefly (10 seconds). Protein concentrations were determined by the Bradford assay (Bio-Rad). Denatured proteins were resolved on a sodium dodecyl sulfate (SDS)-polyacrylamide gels and then transferred onto polyvinyl difluoride membranes. Primary antibodies against mouse HIF-1α (NB100–449; Novus Biological, Littleton, CO) and β-actin (RB-9421, Thermo Scientific) were used for detecting the corresponding proteins. Experiments on NIH3T3 cells, bEND.3 cells, and primary diabetic fibroblasts were performed three times. Densitometry data was calculated using ImageJ software.

### Quantitative reverse transcriptase-polymerase chain reaction (RT-PCR) analysis

Total RNA from treated cells was extracted using the RNeasy Plus Mini Kit (Qiagen) and reverse transcribed by the TaqMan Reverse Transcription Reagents (Applied Biosystems). Real-time quantitative PCR was run on the ABI Prism 7900HT Sequence Detection System (Applied Biosystems) and *Power* SYBR Green Master Mix (Applied Biosystems). PCR was performed by denaturing at 95°C for 15 min, followed by 40 cycles of denaturation at 95°C for 30 s and annealing at 60°C for 1 min. All expression data were normalized to actin. All reactions were run in triplicate. Two independent experiments were performed. The sequences of primers used are listed in **Table S1**.

### Angiogenesis Antibody Array

Proteome Profiler™ Mouse Angiogenesis Antibody Array (R&D Systems) was used according to the manufacturer's protocol. In brief, the nitrocellulose membranes were first incubated in blocking buffer for 1 hr. A cocktail of biotin-labeled antibodies against different individual angiogenesis-related proteins was incubated with about 1 ml of conditioned media prepared from NIH3T3 cells treated with or without 10 µM CPX after normalization with equal amounts of total protein. The sample and antibody mixture was then incubated with the membrane overnight at 4°C. After multiple washings to remove unbounded mateials, the membranes were incubated with horseradish peroxidase (HRP) conjugated streptavidin for 1 hr at room temperature. After washing the membranes, the signals were detected by ECL system (Amersham Pharmacia Biotech Aylesbury, UK). The array image was then semiquantitated by reverse image scanning densitometry (Adobe Photoshop CS4, Adobe Systems). The array was run in duplicates.

### In vitro angiogenesis assay

Approximately 2×10^4^ human umbilical vein endothelial cells (HUVEC) in 50 µl serum free medium were seeded into each well of 96-well plates. The plate wells were coated with 40 µl of BD Matrigel Matrix which was polymerized for 30 min at 37°C. Growth factor (10 ng/ml VEGF) and/or 10 µM of CPX were added to the cell cultures. After 16 hours incubation, the cells were labeled with 8 µg/ml Calcein AM (BD) and were evaluated for capillary tubule formation.

### Wound Model

All experiments were performed in accordance with the Stanford University Animal Care and Use Committee Guidelines (protocol ID #21308). Diabetic *db*/*db* mice (12-15 weeks old, BKS.Cg-*m* +/+ Lepr*^db^*, stock #000642; Jackson Laboratories) were housed five per cage in a 12-h light/dark cycle and provided *ad libitum* with standard food and water.

After depilation, two 6-mm full-thickness wounds extending through the panniculus carnosus were made on dorsa of mice as previously described [13]. A donut-shaped 12 mm silicone splint (Invitrogen) was placed and fixed to the skin with cyanoacrylate glue and interrupted 6–0 nylon sutures so that the wound was centered within the splint. The animals were randomly divided into two groups with 5 animals (and, therefore, 10 wounds) per group. Ten µL of 50 mM CPX or DMSO vehicle was placed topically onto the open wound bed every other day until closure. A Tegaderm (3M) dressing was placed over the wounds after each topical application, and the animals were housed individually. Digital photographs of the wounds were taken every other day until closure. Time to wound closure was defined as the time at which the wound bed was completely re-epithelized and filled with new tissue. Wound area was quantified by tracing the wound margins and calculated as a percent area of the original wound size using ImageJ software (NIH), with scaling normalized to the circular reference of the splint.

To evaluate tissue HIF-1α levels, wounds treated with CPX or DMSO were harvested along with 4-mm rim of normal skin around the wounds on day four post wounding. Unwounded untreated skin was harvested in a similar fashion, with the same diameter of tissue excised. Tissues were minced and homogenized in urea lysis buffer. HIF-1α expression was probed by Western Blot as described above. These experiments were repeated four times.

### Histology, Immunohistochemistry Staining, and Evaluation

After fixation in 4% paraformaldehyde, tissue samples were embedded in optimal cutting temperature (O.C.T.) and sliced into sections of 8 µm thickness using a cryostat. Every tenth section was stained with hematoxylin and eosin (H&E). Four high-powered fields (10×40) of wound areas were chosen from each slide, and total number of cells per field of view was counted. Vascular density was detected using a CD31 monoclonal antibody (1∶500 dilution, BD Biosciences) with a secondary Alexa-Fluor 594-linked anti-rat IgG antibody (1∶1000 dilution, Invitrogen). Vessel density was quantified by assessing total number of red fluorescent-labeled vessels normalized to the entire wound area at 400x magnification, using Image J software. The data represent average values from nine high-power fields from three independent mouse wounds.

### Statistical Analysis

All data are expressed as mean ± SEM. Student's t test or Wilcoxon rank sum tests were used to determine significance. A p value <0.05 was considered statistically significant.

## Supporting Information

Table S1 **Gene primer sequences used for quantitative, real-time polymerase chain reaction (QRT-PCR).**
(DOCX)
