# Supplementary material for: Antimycotic Ciclopirox Olamine in the Diabetic Environment Promotes Angiogenesis and Enhances Wound Healing
Source: PLoS One. 2011 Nov 18;6(11):e27844. doi: 10.1371/journal.pone.0027844 (PMC3220686; doi:10.1371/journal.pone.0027844)
Supplement: Table S1 — Gene primer sequences used for quantitative, real-time polymerase chain reaction (QRT-PCR). (DOCX) [file pone.0027844.s001.docx]

| **Gene Name** | **Primer Sequence (5’ to 3’)** |
| --- | --- |
| FGF-2 | Forward: GTCACGGAAATACTCCAGTTGGT  Reverse: CCCGTTTTGGATCCGAGTTT |
| VEGF | Forward: GCAGAAGTCCCATGAAGTGAT  Reverse: GTCTCAATTGGACGGCAGTAG |
| ANG | Forward: CCAGGCCCGTTGTTCTTGAT  Reverse: GGAAGGGAGACTTGCTCATTC |
| SDF-1 | Forward: TGCATCAGTGACGGTAAACCA  Reverse: TTCTTCAGCCGTGCAACAATC |
| Proliferin | Forward: AGCCCCATGAGATGCAATACT  Reverse: CACTCACTAGATCGTCCAGAGG |
| CX3CL1 | Forward: ACGAAATGCGAAATCATGTGC  Reverse: CTGTGTCGTCTCCAGGACAA |
| CXCL1 | Forward: CTGGGATTCACCTCAAGAACATC  Reverse: CAGGGTCAAGGCAAGCCTC |
| Tissue Factor | Forward: AACCCACCAACTATACCTACACT  Reverse: GTCTGTGAGGTCGCACTCG |
